# A Performance Evaluation of Fly Ash–Plastic Aggregate in Hydraulic Backfilling: A Comparative Study

**DOI:** 10.3390/ma18122751

**Published:** 2025-06-12

**Authors:** Munipala Manohar, Bhanwar Singh Choudhary, Krzysztof Skrzypkowski, Krzysztof Zagórski, Anna Zagórska

**Affiliations:** 1 Department of Mining Engineering, Indian Institute of Technology (Indian School of Mines), Dhanbad 826004, India; manoharmunipala.19dr0093@me.iitism.ac.in; 2 Faculty of Civil Engineering and Resource Management, AGH University of Krakow, Mickiewicza 30 Av., 30-059 Kraków, Poland; skrzypko@agh.edu.pl; 3 Faculty of Mechanical Engineering and Robotics, AGH University of Krakow, Mickiewicza 30 Av., 30-059 Kraków, Poland; zagkrzys@agh.edu.pl; 4 Research Centre in Kraków, Institute of Geological Sciences, Polish Academy of Science, Senacka 1, 31-002 Kraków, Poland; a.zagorska@ingpan.krakow.pl

**Keywords:** fly ash–plastic aggregates, backfill material, hydraulic backfilling

## Abstract

Underground mining creates voids that require filling to prevent ground subsidence and mitigate post-mining issues. Traditionally, sand has been used as the primary backfilling material. However, the increasing demand from the construction sector and the slow natural replenishment of sand have necessitated the search for alternative materials. Researchers have explored fly ash (FA) as a potential substitute; however, its slow settling rate and the development of hydrostatic pressure limit its effectiveness. To address these issues, this study investigated the development of fly ash–plastic aggregate (FPA) as a suitable material for hydraulic backfilling by mixing FA with high-density polyethylene (HDPE) plastic in an 80:20 ratio. Initial investigations revealed that adding plastic as a binder significantly improves the physical, mechanical, and morphological properties of FA. The results further demonstrate that FPA satisfies and exceeds the standard requirements for hydraulic backfilling, as outlined in previous studies and case reports. These findings suggest that FPA is a promising alternative to both sand and FA for hydraulic backfilling applications.

## 1. Introduction

The mining process entails extracting essential minerals required by humans from the Earth, employing methods that ensure safety and economic viability. In underground mining activities, the inevitable consequence of void creation necessitates backfilling to prevent subsidence and to address other post-mining issues. Backfilling is a technique employed to fill the voids left over after the extraction of minerals in underground mining. Experts have recognized this approach as an environmentally acceptable method for waste disposal. The backfilling process is paramount in the comprehensive functioning of mining operations, as it provides appropriate support, minimizes the dilution of waste material, facilitates safe working conditions, and mitigates the potential risk of surface subsidence [1,2]. The materials utilized in this technique are commonly referred to as backfilling material and are categorized into two primary groups, cemented and uncemented [3]. Various factors influence the selection of these materials, including their accessibility, cost, and stowing system and essential properties such as (a) their slake durability (I_d1_ > 95%, I_d2_ > 85%), (b) grain size distribution (silt-sized particles not exceeding 10%), (c) permeability (minimum of 100 mm/h), (d) shear strength properties (angle of internal friction greater than 30°) [1,3], and (e) morphological characteristics [1,4].

For several decades, a prominent method of backfilling has involved the use of sand as an uncemented backfill [5,6]. The imperative to explore alternative materials has emerged because of a scarcity of sand resulting from the huge demand in the construction industry and the decreased replenishment rate emerging from the construction of dams for various purposes. The current focus is on fly ash (FA), a byproduct of coal combustion and a pollutant if stored in ash ponds, as a substitute for sand in backfilling applications. In India, the Ministry of Environment, Forest, and Climate Change (MoEFCC) [6] has also mandated that mines within a 300 km radius of power plants must use FA as a stowing material in underground mines or integrate a minimum of 25% FA with material from external overburdened dumps. Initial studies have indicated that (a) the mineral composition of FA can be augmented with silica and calcium oxide, which facilitates strength development [7,8], (b) its low density enables the pumping of the slurry with reduced pressure and mitigates pipe clogging [9], and (c) the circular morphology of FA provides a ball bearing effect, contributes to friction-less flow, and minimizes the wear of transportation pipes [10]. Additional investigations [11] have indicated that a significant proportion of silt-sized FA impedes the rapid settling of particles, increases the hydrostatic pressure, obstructs layer-like deposition, and leads to inadequate consolidation. Although FA possesses numerous advantageous properties for use as backfill, the significant presence of silt-sized particles requires further investigation into suitable techniques.

At the same time, plastic is one of the most environmentally detrimental substances on the planet. The predominant factors contributing to the excessive utilization of plastic include its affordability, light weight, robustness, corrosion resistance, and exceptional thermal and electrical insulation properties [12]. Consequently, there has been a significant increase in plastic production over the past 70 years. Global plastic production rose from 2 Mt in 1950 to 400 Mt in 2022 [13]. Although plastics are widely utilized for diverse applications, they predominantly end their lifecycle after a single use and become waste. Estimates indicate that global plastic waste generation may increase exponentially from 80 Mt in 2015 to 213 Mt by 2060 [14], and half of this plastic waste ends up in landfills or marine environments [15]. Plastics in landfills decompose with municipal solid waste (MSW) and produce gases. Globally, plastic in MSW produces 3.4 to 3.9% of harmful gases, such as CO_2_, NO, SO_2_, and methane [16].

Recycling is the most effective strategy for mitigating the potential hazards associated with plastic waste. Among the various recycling techniques available, pyrolysis, recasting, and use as binders are the most efficient methods for processing plastic waste [17]. The current study investigated the utilization of plastic as a binder to produce FPA, aiming to address the particle size-related limitations of FA and enhance its effectiveness for backfilling applications. Furthermore, this study aimed to experimentally assess the impact of plastic addition and determine the suitability of FPA as a hydraulic backfilling material. This study involved preparing the aggregates by mixing 80% FA with 20% HDPE plastic to achieve this purpose. The study also evaluated the resulting FPA for key backfilling properties and compared its performance with that of traditional backfill materials, including sand and FA. We also benchmarked its performance against standards for hydraulic backfilling materials established by previous researchers.

## 2. Materials and Methods

### 2.1. Raw Materials and Fly Ash–Plastic Aggregate Preparation

#### 2.1.1. River Sand

**Figure 1:**
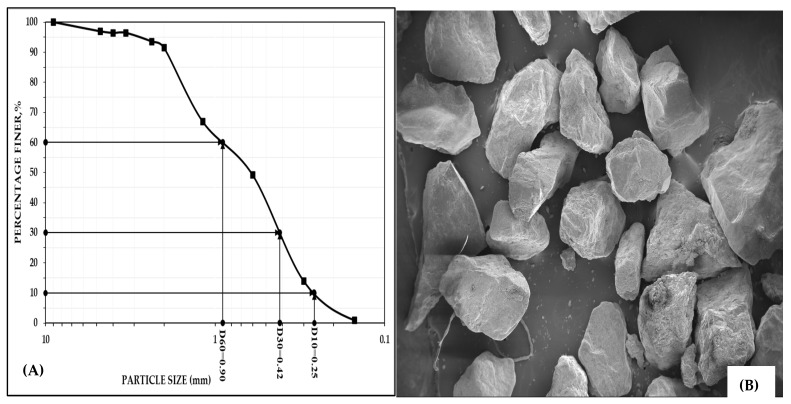
(**A**) Particle size distribution curve; (**B**) scanning electron micrograph of river sand.

For comparative analysis, river sand was procured from the Godavari River in Telangana, India, where it is predominantly used as a backfilling material in mines operated by the Singareni Collieries Company Limited (SCCL) (Kothagudem, India). The geotechnical properties of the sand were evaluated in accordance with the ASTM standards, and the results are presented in Table 1. The particle size distribution curve is shown in Figure 1A and a scanning electron micrograph of the sand is shown in Figure 1B.

The specific gravity of sand was determined to be 2.66. Particle size analysis (PSA) revealed a composition of 3.04% gravel, 5.51% coarse sand, 42.25% medium sand, 48.62% fine sand, and 0.58% silt-sized particles. The sand had an effective particle size (D_10_) of 0.25mm and D_30_ and D_60_ values of 0.42mm and 0.90mm. The coefficient of uniformity (C_u_) was 3.54, and the coefficient of curvature or gradation (C_C_) was 0.77, indicating that the sand was poorly graded. The light compaction test determined the maximum dry density of the sand to be 1.66 g/cc at an optimum moisture content of 5.16%. The permeability of the sand was measured as 1.29 × 10^−4^ m/s. Using X-ray fluorescence (XRF) (Primus IV, Rigaku, Tokyo, Japan), we identified that the major elements present in the sand were 63.03% of SiO_2_, 11.11% of Al_2_O_3_, 5.50% of Fe_2_O_3_, and 13.11% of CaO. The high silica percentage and permeability made the sand a successful stowing material.

#### 2.1.2. Fly Ash

**Figure 2:**
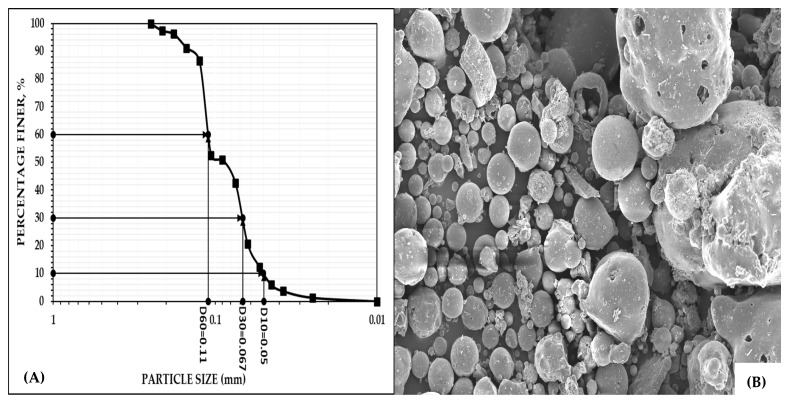
(**A**) Particle size distribution curve; (**B**) scanning electron micrograph of fly ash.

FA was sourced from the Chandrapura Thermal Power Station (CTPS) belonging to Damodar Valley Corporation (DVC) (Kolkata, India). The DVC-CTPS is a 500 MW power plant situated in the town of Chandrapura, the district of Bokaro, Jharkhand State, India, and acquires coal from Central Coalfields Limited (CCL) (Ranchi, India), a subsidiary of Coal India Limited (CIL) (New Town, India). The geotechnical properties of the FA are summarized in Table 1. The particle size distribution curve for the FA and a scanning electron micrograph are shown in Figure 2.

The specific gravity of the FA was determined to be 2.00. The PSA demonstrated a composition of 57.34% fine sand and 42.66% silt-sized particles. The FA had an effective particle size (D_10_) of 50 μm, a D_30_ of 67 μm, D_60_ of 110 μm, a C_U_ of 2.2, and a C_C_ of 0.83, falling into the poorly graded soil category. The light compaction test determined the maximum dry density of the FA to be 1.15 g/cc at an optimum moisture content of 35.3%. The high OMC and low MDD revealed that the particles were small, with a large specific surface area. The permeability of the FA was measured as 1.387 × 10^−6^ m/s, falling into the silt category. Using XRF, we identified the major elements present in the FA as 55.82% SiO_2_, 31.57% Al_2_O_3_, 4.66% Fe_2_O_3_, and 0.65% CaO, meaning it fell into the category of Class F FA. The comparative chemical composition of sand and FA is presented in Table 2.

Although FA has a similar elemental configuration to sand and an advantage over sand in terms of hydraulic transportation due to its lightness, possessing a high percentage of silt-sized particles makes water drainage difficult and causes the development of hydrostatic pressure over the barricades, resulting in their rupture. This functional problem provides an opportunity to explore appropriate ways to modify the material to utilize FA as a successful hydraulic backfilling material.

#### 2.1.3. Plastic

Plastic was used in this study as a binding material to make FPA. High-density polyethylene (HDPE), obtained for the fabrication of FPA, was sourced from the local municipal solid waste collector. The acquired plastic was subjected to a drying process and subsequently shredded into small chips. HDPE has a 950 kg/m^3^ density and a melting point ranging from 130 to 136 °C and crystalizes at 110 °C [18].

#### 2.1.4. Preparation of Fly Ash–Plastic Aggregate

**Figure 3:**
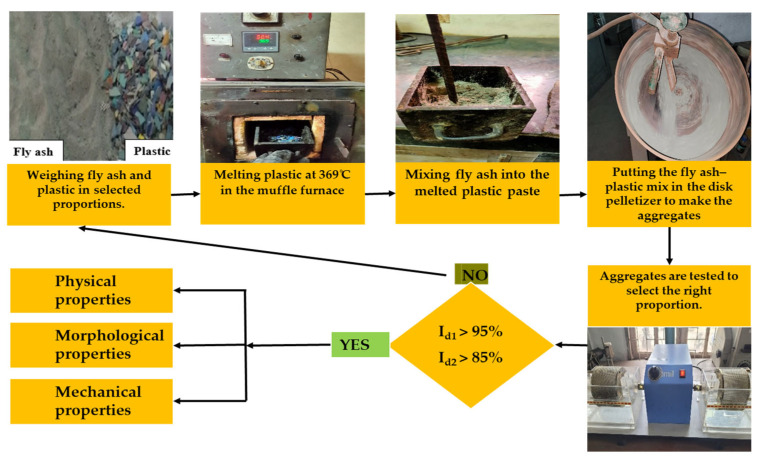
The step-by-step FPA preparation process and sequence of testing.

The preparation began by quantifying the necessary amount of plastic and placing it in a mild steel mold. The mold was subsequently placed in a muffle furnace at 369 °C for 30 min, ensuring the complete melting of the plastic. Upon melting, FA was incorporated during agitation to guarantee an even distribution. After mixing, the molten amalgamation was conveyed to a disk pelletizer inclined at 45° and operating at a rotational speed of 15 rpm to produce aggregates of the desired size. The parameters for the FPA composition, plastic melting temperature, pelletizer angle, and velocity were established based on the initial trials. Aggregates that had been formed were permitted to cool and solidify. The step-by-step FPA preparation process is shown in Figure 3.

### 2.2. Methodology

This study entailed the preparation of FPA and the assessment of its appropriateness for hydraulic backfilling. Firstly, using the slake durability test, FPA was tested for its resistance to disintegration. Aggregate proportions with the minimum required durability (I_d1_ > 95%, I_d2_ > 85%) were tested for their physical, mechanical, and morphological properties. The methodology is shown in Figure 3.

## 3. Results and Discussion

### 3.1. Effect of Plastic Binding on Durability

#### Slake Durability

The slake durability test quantifies fragile rocks’ resistance to weakening and disintegration resulting from cycles of drying and wetting, constituting an index test tailored explicitly for the differentiation of rocks based on their response to wetting and drying. The test was conducted following ASTM D4644 [19].

The test results for FPA, denoted as I_d1_ and I_d2_, with yielded values of 97.09 each, fell under the extremely-high-durability category [20] and surpassed the articulated standards [1]. This high durability signifies that FPA is resistant to considerable disintegration. The durability results from the binding properties of the plastic component, which enables the creation of larger, stable aggregates. This attribute offers an advantage over traditional FA, which does not settle quickly and escapes through barriers. The improved durability of FPA suggests that it may maintain structural integrity during hydraulic transport through pipelines and settle rapidly.

### 3.2. Effect of Plastic Binding on Physical Properties

#### 3.2.1. Particle Size Analysis

**Figure 4:**
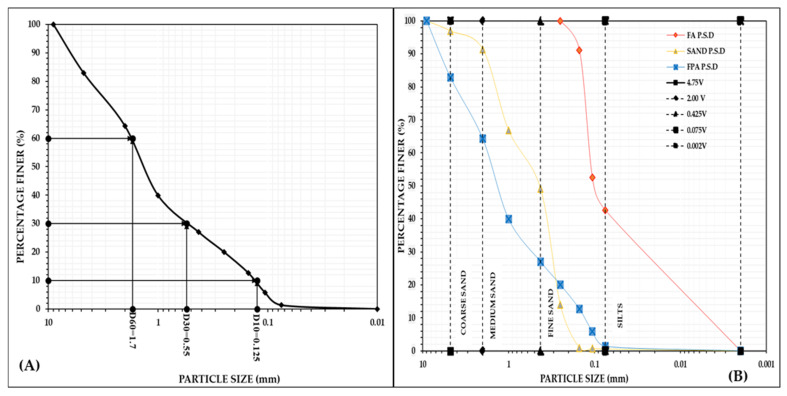
(**A**) Particle size distribution curve of FPA. (**B**) Comparative distribution curves of FPA, FA, and sand.

PSA involves the quantitative categorization of soil particles based on their mass, contributing to the classification of soils. The testing procedure, in adherence with the standard protocol ASTM D2487 [21], facilitated this analysis. Figure 4A illustrates the particle size distribution curve specific to FPA.

The PSA of FPA revealed D_10_ to be 0.125, D_30_ to be 0.55, D_60_ to be 1.7, C_U_ = 13.6, and C_C_ = 1.42, categorizing it as well-graded soil. FPA contained 17.16% gravel, 18.54% coarse sand, 37.28% medium sand, 25.64% fine sand, and 1.38% silt-sized particles. Figure 4B presents the comparative particle size distribution curves of FA, sand, and FPA. It reveals that the distribution curve of FPA is similar to that of sand, containing similar quantities of particles ranging from gravels to silts, unlike FA, which contained only fine sand and silt-sized particles. The comparative percentage distributions of the various-sized particles are given in Table 3.

Using plastic as a binder significantly improved the particle size distribution of FA, which may lead to enhanced settlement, percolation, and water decantation while reducing the risk of barricade clogging. The well-graded nature of the resulting material decreased the void ratio, potentially improving consolidation and reducing compressibility. Notably, the percentage of silt-sized particles (less than 0.075 mm) decreased from 42.66% to just 1.38%; while over 15% of the particles fell within the 0.040 to 0.150 mm range, not more than 20% of the particles were less than 0.1mm in size. These characteristics qualify FPA to be a suitable hydraulic backfilling material, following the criteria outlined in [1,17,18].

#### 3.2.2. Specific Gravity

The specific gravity is defined as the ratio of the mass of solids per unit volume to the mass of an equal volume of water. This dimensionless parameter plays a critical role in determining the suitability of materials for storage, handling, and transportation in backfilling applications. The specific gravity of the materials was determined following ASTM D550 [22].

**Figure 5:**
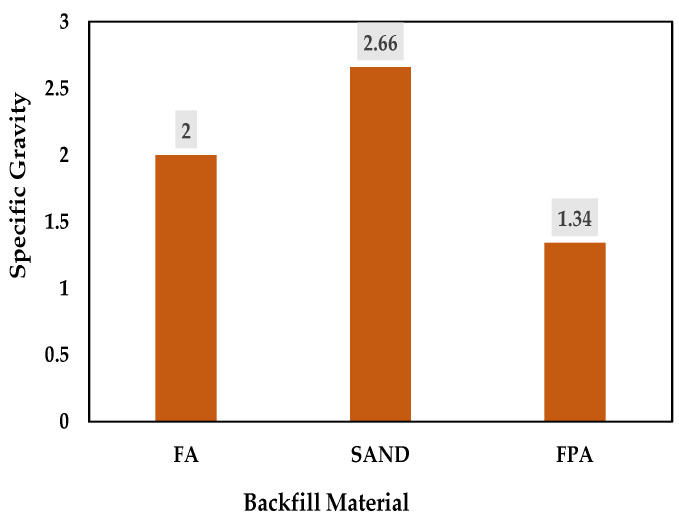
Specific gravity of FA, sand, and FPA.

Incorporating plastic as a binder into fly ash (FA) reduced its specific gravity from 2.00 to 1.34, effectively classifying the resulting FPA as an artificial synthetic lightweight aggregate. A comparative analysis in Figure 5 clearly shows that FPA possessed a significantly lower specific gravity compared to both FA and sand. This reduction in specific gravity offers a practical advantage in hydraulic backfilling operations, as the lighter material can be pumped more easily with a reduced risk of clogging, even with low-pumping heads [17,20].

#### 3.2.3. Permeability

Permeability is the ease of the water flow through the soil pores [23], which holds significance in selecting hydraulic backfill materials [24]. The constant head permeability test measured the permeability of sand and FPA, and the falling head test was used for FA. The test was conducted as per the ASTM D2434 [25].

**Figure 6:**
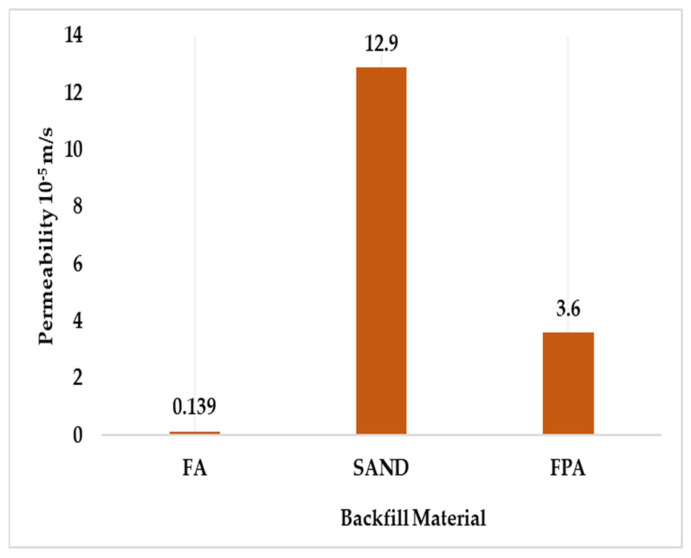
Permeability of FA, sand, and FPA (10^−5^ m/s).

This study assessed the permeability of FA and sand as being 1.39 × 10^−6^ m/s (5 mm/h) and 1.29 × 10^−4^ m/s (464.4 mm/h), respectively. Incorporating plastic with FA resulted in an FPA with a permeability of 3.6 × 10^−5^ m/s (129.6 mm/h). This indicates that using plastic as a binder increased the permeability of FA by approximately 25 times. Although FPA contains a high percentage of gravel- and sand-sized particles, its well-graded nature contributes to a reduced void ratio, resulting in it having a lower permeability than sand. However, it still outperformed the hydraulic backfill material permeability standard of 100 mm/h. Figure 6 gives the comparative permeability values.

Adequate permeability results in rapid water drainage, preventing hydrostatic pressure development, promoting better settlement and consolidation [10,26,27], and eliminating the possibility of pore pressure development, the liquefaction of material, and the rupturing of barricades [10,28].

### 3.3. Effect of Plastic Binding on Morphology

#### Scanning Electron Micrograph Analysis

**Figure 7:**
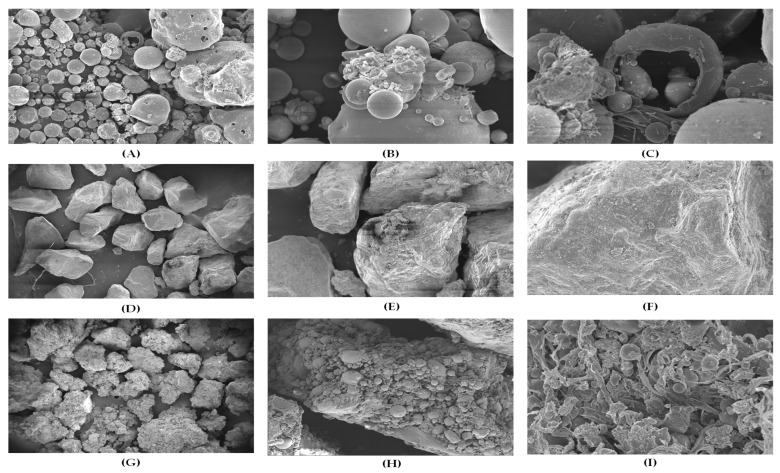
FESEM micrographs of FA (**A**–**C**), sand (**D**–**F**), and FPA (**G**–**I**).

Morphological analysis was conducted to evaluate and compare the particle size, shape, and surface texture of FA, sand, and FPA to assess their influence on relevant geotechnical properties. In the present study, the samples were coated with platinum and analyzed using an FE-SEM Supra 55 (Carl Zeiss, Oberkochen, Germany) instrument equipped with a secondary electron (SE) detector (Carl Zeiss, Oberkochen, Germany). FESEM images were captured at magnifications of 100×, 1000×, and 2000×. The micrographs for FA (A, B, C), sand (D, E, F), and FPA (G, H, I) are presented in Figure 7.

Figure 7A–C show that the FA primarily consisted of round-shaped particles with smooth surfaces, accompanied by a minor presence of elongated, thin, plate-like, or flaky particles. This morphological characteristic suggests the presence of clay-sized fractions to a limited extent. Additionally, the FA also contained hollow particles (cenospheres), as well as hollow particles encapsulating smaller particles (plerospheres). Figure 7D–F reveal that the sand particles exhibited sub-angular to angular and shell-shaped particles characterized by irregular outlines, sharp edges, and relatively smooth textures.

The influence of the plastic binder is evident in Figure 7G–I, where the combination of sand and FA particles formed sand-like angular shapes with a combination of smooth, rough, and jagged surface textures, resembling the characteristic features of a breccia-like structure. Figure 7I provides a sectional view of the FPA, clearly illustrating how effectively the plastic binder held the FA particles together, resulting in a well-structured composite.

Based on the PSA and morphological observations, it is evident that the FPA contained a considerable proportion of coarse-sized, angular particles and exhibited well-graded distribution characteristics that were strong indicators of a high-shear-strength soil [29].

### 3.4. Effect of Plastic Binding on Mechanical Properties

**Figure 8:**
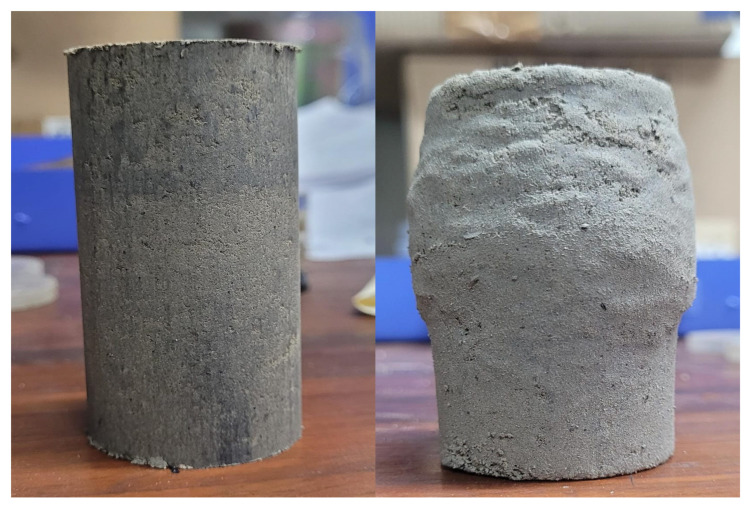
FPA sample before and after the triaxial test.

The Unconsolidated Undrained (UU) triaxial test was performed to assess the mechanical behavior and shear strength parameters (cohesion (c) and the internal friction angle (F)). The testing procedure adhered to the specifications delineated in ASTM D2850 [30]. Stress–strain curves at different confining pressures (50 kPa, 100 kPa, and 150 kPa) were plotted to evaluate the mechanical behavior of the materials. The maximum shear strength values recorded at each confining pressure were utilized to create shear strength envelopes, from which the shear strength parameters were subsequently obtained. A sample before and after the triaxial test is presented in Figure 8.

#### 3.4.1. Stress–Strain Behavior

**Figure 9:**
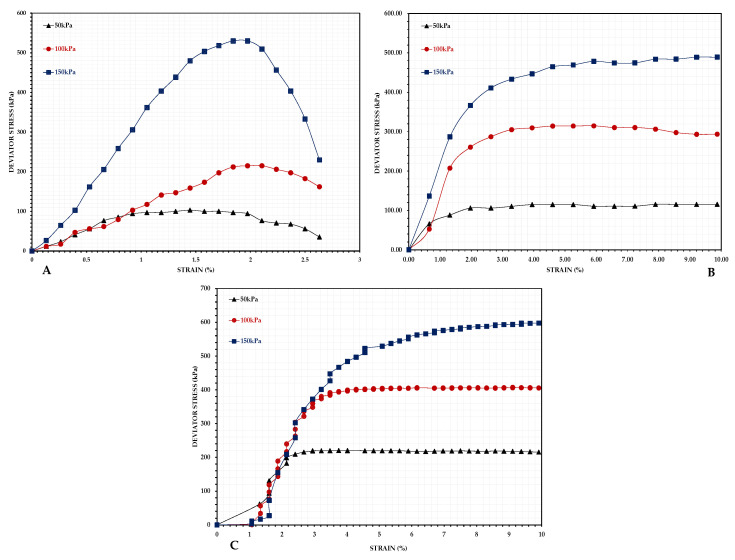
Stress–strain behavior curves of (**A**) FA, (**B**) sand, and (**C**) FPA.

Figure 9A illustrates the stress–strain curves for the fly ash (FA), demonstrating a peak strength of around 530 kPa at a strain of under 2%, succeeded by a rapid decline in stress. This response demonstrates characteristics of brittle failure and strain-softening behavior. The FA exhibited significant sensitivity to the confining pressure, with its strength increasing in response to an elevated confining pressure. Figure 9B demonstrates the behavior of the sand, achieving a peak strength of around 490 kPa without a clear peak, signifying strain-hardening behavior. The residual strength was near the peak strength, indicating stable performance after the peak. Figure 9C illustrates the stress–strain behavior of the FPA. The curves demonstrate an initial steep increase, followed by a gradual transition, ultimately achieving a peak strength of around 600 kPa. The ongoing rise in the stress with the strain indicates a strain-hardening response, with the residual strength remaining near the peak, signifying stable mechanical behavior.

The stress–strain analysis indicated that the FA experienced brittle failure, leaving it unsuitable for backfilling applications. The strain-hardening behavior of the FPA, paired with its similar residual strength to that of sand, indicates an enhanced load-bearing capacity [31]. This suggests that FPA may serve effectively as a backfilling material, thereby improving the stability of the overlying strata [32].

#### 3.4.2. Shear Strength Parameters

**Figure 10:**
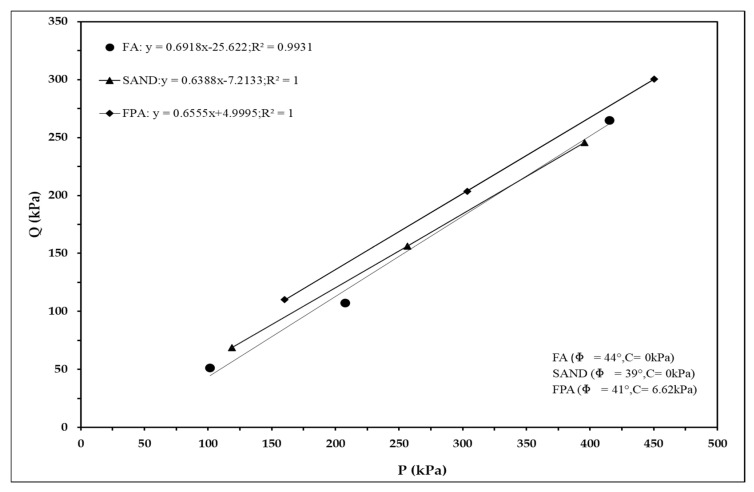
Combined maximum shear stress failure envelope plots of FA, sand, and FPA.

To assess the UU shear strength parameters of the materials, maximum shear stress failure envelope plots were drawn using the P-Q values presented in Table 4, as shown in Figure 10. The results indicate that both the FA and sand exhibited no apparent cohesion, while the F was higher for the FA compared to the sand. The FPA demonstrated a higher internal friction angle than the sand and exhibited a small degree of cohesion.

Although FA consists of fine, round, and smooth particles with poor gradation, its high internal friction angle may be attributed to over-consolidation and the influence of the high confining pressure [33,34]. The developed FPA, which exhibited a particle shape similar to that of sand, showed an internal friction angle that was comparable to that of sand and demonstrated slight cohesion. This may have been due to normal consolidation and enhanced particle interlocking, facilitated by its rough and jagged surface texture [34].

## 4. Conclusions

The investigation into the utilization of fly ash–plastic aggregate (FPA) as a hydraulic backfilling material, developed through the combination of 80% fly ash (FA) and 20% high-density polyethylene (HDPE) plastic, led to the following results:

- The slake durability index of FPA was recorded as I_d1_ = I_d2_ = 97.09, classifying it within the category of extremely high durability.
- Particle size distribution analysis showed that FPA contains an increased proportion of gravel- and sand-sized particles, with less than 10% silt-sized particles, and exhibits a well-graded distribution.
- The specific gravity of FPA was measured at 1.34, making it lighter than both FA and sand.
- The permeability of FPA was found to be 3.6 × 10^−5^ m/s, which is approximately 25 times greater than that of FA.
- FESEM analysis revealed that FPA exhibits sand-like, angular particle morphology with smooth edges and a rough surface texture.
- The stress–strain behavior indicated that FPA undergoes strain hardening without a distinct peak, similarly to sand and in contrast to the brittle or strain-softening behavior observed in FA.
- Shear strength analysis showed that FPA has an internal friction angle of 40°57′ and exhibits a small degree of cohesion.

In conclusion, the 80:20 proportion of FPA, having met the primary slake durability index criteria for backfill applications, was deemed suitable and thus selected for the comparative analysis. This comparative study established that FPA possesses enhanced properties compared to FA and exceeds the standard requirements for hydraulic backfilling properties outlined in previous research. These findings position FPA as a promising alternative material to sand and FA for hydraulic backfilling applications.

## Figures and Tables

**Table 1:** Geotechnical properties of river sand and fly ash.

Property	River Sand	Fly Ash
Specific gravity	2.66	2.00
Grain size analysis	D_10_ = 0.26	D_10_ = 0.032
	D_30_ = 0.41	D_30_ = 0.044
	D_60_ = 0.9	D_60_ = 0.075
	C_U_ = 3.46	C_U_ = 3.46
	C_C_ = 0.72	C_C_ = 0.72
	Poorly graded	Poorly graded
Optimum moisture content	5.16%	35.3%
Maximum dry density	1.66 g/cc	1.15 g/cc
Coefficient of permeability	1.29 × 10^−4^ m/s	1.387 × 10^−6^ m/s

**Table 2:** Chemical composition of river sand and fly ash.

Material	Chemical Composition										
	SiO_2_	Al_2_O_3_	Fe_2_O_3_	CaO	TiO_2_	MnO	MgO	K_2_O	Na_2_O	P_2_O_5_	LOI
River sand	63.03	11.11	5.50	13.11	1.62	0.10	1.27	2.77	1.31	0.17	0.00
Fly ash	55.82	31.57	4.66	0.65	2.36	0.04	0.37	1.51	0.06	0.45	2.50

**Table 3:** Percentage distribution of particles.

Material	Gravel (4.75 to 20 mm)	Coarse Sand (2 to 4.75 mm)	Medium Sand (0.425 to 2 mm)	Fine Sand (0.075 to 0.425 mm)	Silts (0.002 to 0.075 mm)
FA	0	0	0	57.34	42.66
Sand	3.04	5.51	42.25	48.62	0.58
FPA	17.16	18.54	37.28	25.64	1.38

**Table 4:** Triaxial data for plotting maximum shear stress failure envelopes.

Material	(σ_3_) kPa	(σ_1_) kPa	P (kPa) (σ_1_ + σ_3_)/2	Q (kPa) (σ_1_ − σ_3_)/2	Regression Equation of Failure Envelope	Cohesion (kPa)	Angle of Internal Friction (F)
Fly Ash	50	153.00	101.50	51.50	y = 0.6918x − 25.622 R^2^ = 0.9931	0	43°46′
	100	314.98	207.49	107.49			
	150	680.02	415.01	265.01			
Sand	50	187.36	118.67	68.67	y = 0.6388x − 7.2133 R^2^ = 1	0	39°42′
	100	412.95	256.47	156.47			
	150	641.09	395.54	245.54			
FPA	50	270.39	160.19	110.19	y = 0.6555x + 4.9995 R^2^ = 1	6.62	40°57′
	100	507.28	303.64	203.64			
	150	750.85	450.43	300.43			

## Data Availability

The original contributions presented in this study are included in the paper; further inquiries can be directed to the corresponding author.

## Abbreviations

The following abbreviations are used in this manuscript:

FA	Fly Ash
FPA	Fly Ash–Plastic Aggregate
HDPE	High-Density Polyethylene
MoEFCC	Ministry of Environment, Forest and Climate Change
MSW	Municipal Solid Waste
SCCL	Singareni Collieries Company Limited
ASTM	American Standards for Testing Materials
PSA	Particle Size Analysis
C_U_	Coefficient of Uniformity
C_C_	Coefficient of Curvature
XRF	X-Ray Fluorescence
OMC	Optimum Moisture Content
MDD	Maximum Dry Density
CTPS	Chandrapura Thermal Power Station
DVC	Damodar Valley Corporation
FESEM	Field Emission Scanning Electron Microscopy
SE	Secondary Electron
C	Cohesion
F	Angle of Internal Friction
